# Dermatology

**Published:** 1896-09

**Authors:** Henry Waldo


### DERMATOLOGY.

Mr. Hutchinson [1] says that no forms of **eczematous inflammation** ever tend to spontaneous recovery, but unless treated become more extensive and more severe. The exceptions to

[1] *Clin. J.*, 1895, vi. 275.

this are when the patient changes place of residence or mode of life. He thinks proof is abundant and convincing that the tendency to eczema may be hereditary. Thus he says it may prevail in several persons in the same family, and may occur with greater severity in successive generations. The laws under which it is transmitted appear to him to be the same as those of other heritable diseases. They do not prove that the disease, as a totality, is inherited, but rather, perhaps, that a condition of skin especially liable to it is so transmitted. Mr. Hutchinson considers the eczema of infants has nothing whatever to do with the child's health. The infant is usually quite well up to the time of its commencement, and excepting in so far that the dermatitis may interfere with its comfort and rest it remains so throughout. The disease is somewhat difficult to cure until about the end of the lactation period. Even in the most troublesome cases medical treatment generally succeeds about the time that the child is being put upon a mixed diet, and is fed less exclusively on milk. Mr. Hutchinson infers that the dermatitis when at its height is due to the existence of some contagious material which has in some way been generated, and that it is only in a very feeble sense of the words a constitutional malady. It may be observed respecting some cases of general eczema in senile persons that it is very difficult to distinguish them from the disease known as pityriasis rubra, or dermatitis universalis. In the whole domain of eczema we must keep clearly in mind the fact that individual peculiarities in the organisation of the skin are the fundamental predisposing cause. Mr. Hutchinson says eczema in association with acute attacks of gout and the formation of tophi is undoubtedly very rare. He remembers very few instances indeed in which he has met the two conditions together. He is quite prepared to admit, however, that certain articles of diet, such as milk, sugar, and some kinds of fruit, may have the effect of making the skin irritable, and thus increasing the tendency to eczema. Thus it may in some cases be advisable to regulate the patient's diet. In the majority of cases, however, nothing can be done by diet or drugs for the cure of chronic eczema or for the prevention of acute attacks, and to local treatment must we pay especial attention. That there is any one microbe which is the cause of the eczematous process in general appears to Mr. Hutchinson, in the light of clinical facts, improbable in the highest degree.

Dr. Allan Jamieson[1] thinks it is somewhat singular that **disorders of the vermilion portion of the lips** receive in general but scant notice in most works on dermatology. The best known of these is the common herpes labialis, so often met with in association with an ordinary catarrhal attack, or in some febrile conditions with the subsidence of the pyrexial symptoms, but

[1] *Brit. M. J.*, 1895, ii. 1410.

occurring also quite independently of any such state. Painting the erythema or vesicles, as soon as they appear, with a film of flexible collodion checks the further progress and expedites the cure. In those of the male sex the protection of the beard and moustache when worn is a valuable prophylactic. Another and more troublesome affection of the lips is the formation of a small though deep and painful fissure in the centre of the lower lip, where the two halves from which it is developed become fused into one, or at the angles of the mouth, or, but less commonly, in the upper lip, on either side of the central portion of the three parts from the coalescence of which it is formed. The treatment of the fissures in the centre of the lip consists in scraping the whole surface carefully but thoroughly with a fine curette; then, when the bleeding has ceased, having approximated the edges by pressure, a film of salicylic wool is placed on and secured in place by painting over with flexible collodion. Great care must be enjoined to avoid stretching the part till firm union has been secured. Thus a very small spoon should be used, the morsels introduced into the mouth must be restricted in size, while fluids ought to be imbibed through a feeding cup. Those at the angles of the mouth, if antisyphilitic remedies are not called for, can usually be cured by cleanliness and the employment of an antiseptic ointment, such as 10 grains of salicylic acid in 1 ounce of cold cream. Eczema limited to the exposed portions of the lips is comparatively rare. The crusts should be softened and removed by aid of boric starch poultices applied cold. Strips of Unna's zinc ichthyol salve muslin should be closely and constantly laid on at night, the salicylic cold cream already mentioned repeatedly smeared on during the day, and, if anæmia is present, iron prescribed for internal use.

In his introductory address as President of the Dermatological Society of Great Britain and Ireland, Dr. Joseph Frank Payne[1] said the most important problem relating to diseases of the skin is undoubtedly that of causation or etiology. The only branch of the **etiology** of diseases of the skin which can be discussed now is the action of **micro-organisms.** Many parts of the healthy skin harbour bacteria in immense numbers; but the problem arises whether they are merely more or less harmless saprophytes, or in any way pathogenic. In various morbid conditions of the skin, also, bacteria have been found; and here again the question is whether they are really pathogenic. Among the forms most frequently met with are micrococci. Certain species of these—viz., Staphylococcus pyogenes aureus, S. albus, and the rarer S. citreus—occur in many suppurative affections of the skin, as impetigo, ecthyma, pustular acne, folliculitis, sycosis, boils, carbuncles, whitlows, and so on. There can be no doubt that these organisms are really the cause

[1] *Brit. J. Dermat.*, 1896, viii. 267.

of suppuration, though other agencies co-operate. It is, however, to be remembered that these staphylococci are often found as saprophytes in various parts of the skin, as in moist surfaces between the toes, dirt under the nails, scurf on the scalp and elsewhere. They are, in fact, widely present in or on the human skin, without requiring to be introduced from without; and though sometimes present, as it seems, in dwellings and on clothing, have not an important ectogenic existence. Hence external septic infection as producing suppuration is now recognised as a less serious risk in surgery than that of personal contagion. Dr. Payne asked, What is the difference between the highly contagious character of impetigo contagiosa, which approaches that of a specific disease, and the inert saprophytic character of the staphylococci in other cases? The difference depends upon a gradual evolution of virulence, which may be clearly traced in some cases. For instance, a healthy young person may have a wound on the foot. This, influenced by the saprophytic cocci of the part, suppurates. The cocci cultivated in this local inflammation become sufficiently virulent to produce vesicles and pustules in the neighbourhood. Transferred thence by the finger-nails to the face, a classical impetigo contagiosa, with inflamed glands, may result, which precisely resembles an impetigo conveyed by inoculation from another person. This may in certain cases give rise to contagion, spreading to another individual, since cultivation with abundant access of air seems to give these aerobic organisms increased virulence. Finally, transmission from one case to another in rapid succession, according to a well-known pathological law, still further heightens their virulence, till at length the acute, contagious, and apparently specific disease results. The same progressive evolution of virulent properties may also, it should be noticed, be traced in other cases. For instance, the pneumococcus and other bacteria of pneumonia are occasionally found in the mouths of healthy persons, without producing disease, and the same is true of the bacillus diphtheriæ, which, however, if transferred to another person, may be highly pathogenic. The gradual evolution of epidemics of diphtheria out of what is called a simple sore-throat may also receive a similar explanation.

Of the **etiology of eczema** Dr. Payne inclines to the view that there are microbes originally present in the skin, and that when their activity is heightened by some trauma, or disturbance of nutrition, eczema results. It rarely happens that the microbes of eczema acquire such virulence as to produce actual contagion to another individual. In a disease so multiform as eczema it is probable that several species of bacteria may be concerned.

An inquiry into the **plurality of fungi causing ringworm** in human beings as met with in London has been undertaken by Drs. Colcott Fox and Blaxall.[3] The material was collected

[3] *Brit. J. Dermat.*, 1896, viii. 241.

from each case in small bottles of ether. The ether was subsequently poured off and replaced by liquor potassæ (B. Ph.) of about 7 per cent. strength. Sabouraud recommends that hairs should be placed in liquor potassæ of 40 per cent. strength, and then carefully warmed. The softened specimens are floated on to the slide, and care taken not to disarrange the position of the fungus by pressure of the cover-glass. The clinical appearances were carefully noted, and an endeavour made to trace the source of infection. Cultures were also obtained by the inoculation of suitable media. They agree with Sabouraud that the ringworms met with in human beings comprise two affections caused by fungi quite distinct from one another, clinically, morphologically, culturally, and botanically. The more frequent one is caused by the *Microsporum Audouini*. The less frequent one is caused by various species of *Trichophyton* fungus.

The **Microsporum** is a ringworm with small spores, which vary in size within certain limits, are primarily round or oval, and become polyhedral by pressure. It is an affection of early childhood; invariably respecting the scalp of adults. It never produces sycosis of the beard, and does not attack the nails. It is the most extensive, contagious, and rebellious of scalp ringworms, and the most frequently epidemic. The hairs in the patch are all broken off at different lengths, forming relatively long stumps, which are slender, decolorised, greyish, and break off within the follicle when pulled. They possess a white coating for a little distance beyond the follicle. The hair itself is filled with extremely delicate parallel mycelial threads, of which the largest, and also the central, ones have delicate septa at long intervals.

The **Trichophyton** comprises the ringworms with comparatively large spores. They are divisible into two great groups: the one apparently of exclusively human origin, characterised by a marked tendency to affect only the interior of the hair when that structure is attacked, and hence denominated *Trichophyton megalosporon endothrix;* the other apparently of animal origin, and locating itself chiefly between the wall of the follicle and the hair, as well as entering the shaft, and hence called *Trichophyton megalosporon ectothrix* or *endo-ectothrix*. The *endothrix* fungus may occur rather later in childhood than the *microsporum* ringworm, and is on the whole not so persistent. It has not been found to produce a parasitic sycosis of the beard, nor does it attack the nails. The glabrous skin is, however, frequently involved, and circinate lesions result. Family epidemics occur. As in all trichophytic scalp-lesions, an epidemic circination precedes the parasitic invasion of the hair, but is fugacious, and little marked. The hairs are much swollen, darkened in colour, broken off very short, and are without a pseudo-epidermic, white parasitic sheath. The hairs break off on a level with the skin here and there, and the surface of the patch is riddled with black points like comedones. Adults may be affected. The fungus consists of mycelial filaments, composed of double-contoured cells (except one ovoid spore variety), which are the mycelial spores, joined end to end. These sporulated threads are closely packed side by side *within* the whole extent of the diseased stump. According to Sabouraud, the second group of the Trichophyton family is composed of the varieties of *Trichophyton megalosporon ectothrix*, which are, as already stated, of animal origin. They are responsible for a very small minority of scalp ringworms—perhaps 5 per cent.—but for all beard sycosis of the ringworm order, and for ringworm of the nails. They cause more than half the cases of tinea circinata, and, reckoning all sites, about half of the trichophytic ringworms. The lesions from these are all readily cured in a few months. The fungus is found on microscopical examination to be limited to the intrafollicular region, or rarely to affect the hair itself, hence the term "endo-ectothrix." As in all the trichophytons, the spores are agminated in chains, but the sporulation is less regular than in endothrix. Sabouraud some time ago had isolated fourteen species of *ectothrix trichophyton* in two hundred and thirty cases consecutively examined. The authors of this paper find that the fungus answering clinically and microscopically to *microsporum* causes something like 80 to 90 per cent. of ringworms met with in London. Sabouraud believes that the scalp is implicated secondarily to the hair when the fungus is *Microsporum Audouini*, and in this contrasting with the trichophytons. The authors of this paper disagree with this view. They say if the skin of a ringworm patch of the scalp be examined in the early spreading stages there is always a quantity of mycelial feltwork present, even before the hairs are appreciably affected, and that this tinea circinata character disappears *pari passu* with infection of the hairs. They agree with Adamson on this point.

Dr. James Galloway[1] says the word lupus has come to bear so distinctly the tubercular stamp that most will agree that it is a pity that **lupus erythematosus** ever received the name. Almost universally the latter disease is considered to be a completely separate entity, and to have nothing in common with tuberculosis of the skin. Its relations to conditions produced by defective circulation, especially to diseases of the chilblain group, have long been emphasised by English authorities.

Dr. Brocq[2] quotes the case of one of his own patients who for years suffered from severe lupus vulgaris (non-exedens) which resisted all forms of local and general treatment. This patient also suffered from recurrent attacks of lupus erythematosus of the fingers, which was controlled by the exhibition of iodoform. Death occurred, however, preceded by symptoms exactly resembling those produced by Koch's tuberculin. Brocq bases the opinion that in a focus of tuberculosis—*e.g.* lupus vulgaris—

[1] *Practitioner*, 1895, lv. 493.
[2] *J. Cutan. & Genito-Urin. Dis.*, [1895], xiii. 345.

toxins may be elaborated which on absorption produce conditions of erythema, and that one of these is lupus erythematosus. Brocq would thus include lupus erythematosus in the same category as urticaria and the symptomatic erythemata. In support of his views he brings forward the numerous cases of transient lupus erythematosus, and other cases in which large areas of erythema appear and gradually vanish, leaving only the central atrophic portion of skin so typical of this affection. He also suggests that lupus erythematosus may possibly be caused by poison in the circulation, causing vaso-motor paralysis, in certain cases possibly absorbed from foci of tubercular disease.

HENRY WALDO.

